# Establishment of a heart-on-a-chip microdevice based on human iPS cells for the evaluation of human heart tissue function

**DOI:** 10.1038/s41598-020-76062-w

**Published:** 2020-11-05

**Authors:** Mosha Abulaiti, Yaxiaer Yalikun, Kozue Murata, Asako Sato, Mustafa M. Sami, Yuko Sasaki, Yasue Fujiwara, Kenji Minatoya, Yuji Shiba, Yo Tanaka, Hidetoshi Masumoto

**Affiliations:** 1 Clinical Translational Research Program, RIKEN Center for Biosystems Dynamics Research, 2-2-3 Minatojima Minami-machi, Chuo-Ku, Kobe, 650-0047 Japan; 2 Department of Cardiovascular Surgery, Graduate School of Medicine, Kyoto University, Kyoto, Japan; 3 Laboratory for Integrated Biodevice, RIKEN Center for Biosystems Dynamics Research, Suita, Japan; 4 Institute for Advancement of Clinical and Translational Science, Kyoto University Hospital, Kyoto, Japan; 5 Laboratory for Morphogenetic Signaling, RIKEN Center for Biosystems Dynamics Research, Kobe, Japan; 6 Department of Regenerative Science and Medicine, Institute for Biomedical Sciences, Shinshu University, Matsumoto, Japan

**Keywords:** Cardiovascular diseases, Induced pluripotent stem cells, Biomedical engineering

## Abstract

Human iPS cell (iPSC)-derived cardiomyocytes (CMs) hold promise for drug discovery for heart diseases and cardiac toxicity tests. To utilize human iPSC-derived CMs, the establishment of three-dimensional (3D) heart tissues from iPSC-derived CMs and other heart cells, and a sensitive bioassay system to depict physiological heart function are anticipated. We have developed a heart-on-a-chip microdevice (HMD) as a novel system consisting of dynamic culture-based 3D cardiac microtissues derived from human iPSCs and microelectromechanical system (MEMS)-based microfluidic chips. The HMDs could visualize the kinetics of cardiac microtissue pulsations by monitoring particle displacement, which enabled us to quantify the physiological parameters, including fluidic output, pressure, and force. The HMDs demonstrated a strong correlation between particle displacement and the frequency of external electrical stimulation. The transition patterns were validated by a previously reported versatile video-based system to evaluate contractile function. The patterns are also consistent with oscillations of intracellular calcium ion concentration of CMs, which is a fundamental biological component of CM contraction. The HMDs showed a pharmacological response to isoproterenol, a β-adrenoceptor agonist, that resulted in a strong correlation between beating rate and particle displacement. Thus, we have validated the basic performance of HMDs as a resource for human iPSC-based pharmacological investigations.

## Introduction

Heart disease is the greatest cause of death worldwide^1^. The current standard treatment of heart failure includes administration of β-adrenergic receptor blockers and angiotensin receptor blockers, for which the advantageous effects on the prevention of death and major adverse cardiovascular and cerebrovascular events have been proven by large-cohort clinical studies^2,3^. However, the effects of these drugs on heart failure in advanced stages are still limited^4,5^. Development of therapeutic drugs effective for the amelioration of clinical outcomes for severe heart failure mainly due to ischaemic or dilated cardiomyopathies is anticipated.

The history of modern drug discovery has proven that cardiac toxicity is the most critical event in new drug development and has been a common cause of the withdrawal of drugs from the market^6,7^. From 1990–2001, eight drugs with noncardiovascular targets were withdrawn due to cardiac toxicity, which resulted in estimated medical expenditures of $12 billion in total^8^. We should remember that those agents had successfully passed standardized safety tests of those days before clinical use, indicating that more precise detection of cardiac side effects in earlier stages of drug development is indispensable to avoid irremediable damage to drug discovery. In addition to in vivo animal tests with telemetry, the guideline advocates the use of mammalian cell lines that constitutively overexpress the human ether-a-go-go related gene (hERG) encoding the cardiac delayed-rectifying K^+^ (IKr) channel^9,10^. However, it has also been reported that screening of drugs based on the hERG test, which blocks a single ion channel in non-human mammalian cells, can be associated with false negatives (i.e., alfuzosin) and false positives (i.e., verapamil)^11^, leading to the release of potentially lethal drugs to the market and/or the attrition of the use of beneficial drugs.

Human induced pluripotent stem cells (iPSCs) have recently attempted to be used for drug discovery and drug safety tests in various target organs as a resource of human somatic cells^12,13^. Recent studies of drug safety tests using human iPSC-derived cardiomyocytes (CMs) opened a gate to use human cells that show greater fidelity than those used in hERG tests^14,15^. Nevertheless, the substantial limitation of the methods based on single cells is that they can only detect phenomena occurring in single cells per se^16^ and still fail to show the actual kinetics of native myocardial tissues resulting from the interaction of multiple cells in heart tissue, which is composed of not only CMs but also other cell lineages such as vascular cells and stromal cells.

Biomimetic human heart tissue-like structures composed of various cardiac cell lineages would be desirable for more precise evaluations of physiological heart function in response to candidate drugs. We have been investigating biomimetic cardiac tissue sheets as cardiac microtissues derived from human iPSCs that are composed of various cardiovascular cells using temperature-responsive culture dishes as a heart tissue surrogate to recapitulate human heart tissue function, which would serve as an optimal resource for preclinical drug discovery and safety tests^17^.

To apply biomimetic human heart tissue-like structures, such as the aforementioned human iPSC-derived cardiac microtissues, to drug discovery and cardiac toxicity tests, it is indispensable to develop a bioassay system to convert the small pulsations of cardiac microtissues into indicators of tissue function with higher sensitivity and versatility compared to those of previously reported systems based on cantilever or force measurement devices^18–20^. Organ-on-a-chip is an emerging concept to recapitulate organ function using polymeric organosilicon compounds such as polydimethylsiloxane (PDMS) by utilizing Micro Electro Mechanical Systems (MEMS) technology, which would be applied for the establishment of the bioassay system^21–23^. MEMS-based organ-on-a-chip technology potentially facilitates the establishment of microdevices recapitulating heart pump function as a highly sensitive bioassay system for drug discovery and cardiac toxicity tests.

In the present study, we developed a heart-on-a-chip microdevice (HMD) as a novel bioassay system to evaluate the tissue function of human iPSC-derived cardiac microtissues by integrating two fundamental technologies, MEMS-based organ-on-a-chip technology and human iPSC technology, and we hypothesized that the HMD recapitulates heart tissue function by validating the ability of the system to respond to electrical stimulation and dose-dependent inotropic drug administration.

## Results

### Preparation of human iPSC-derived 3D cardiac microtissues

**Figure 1:**
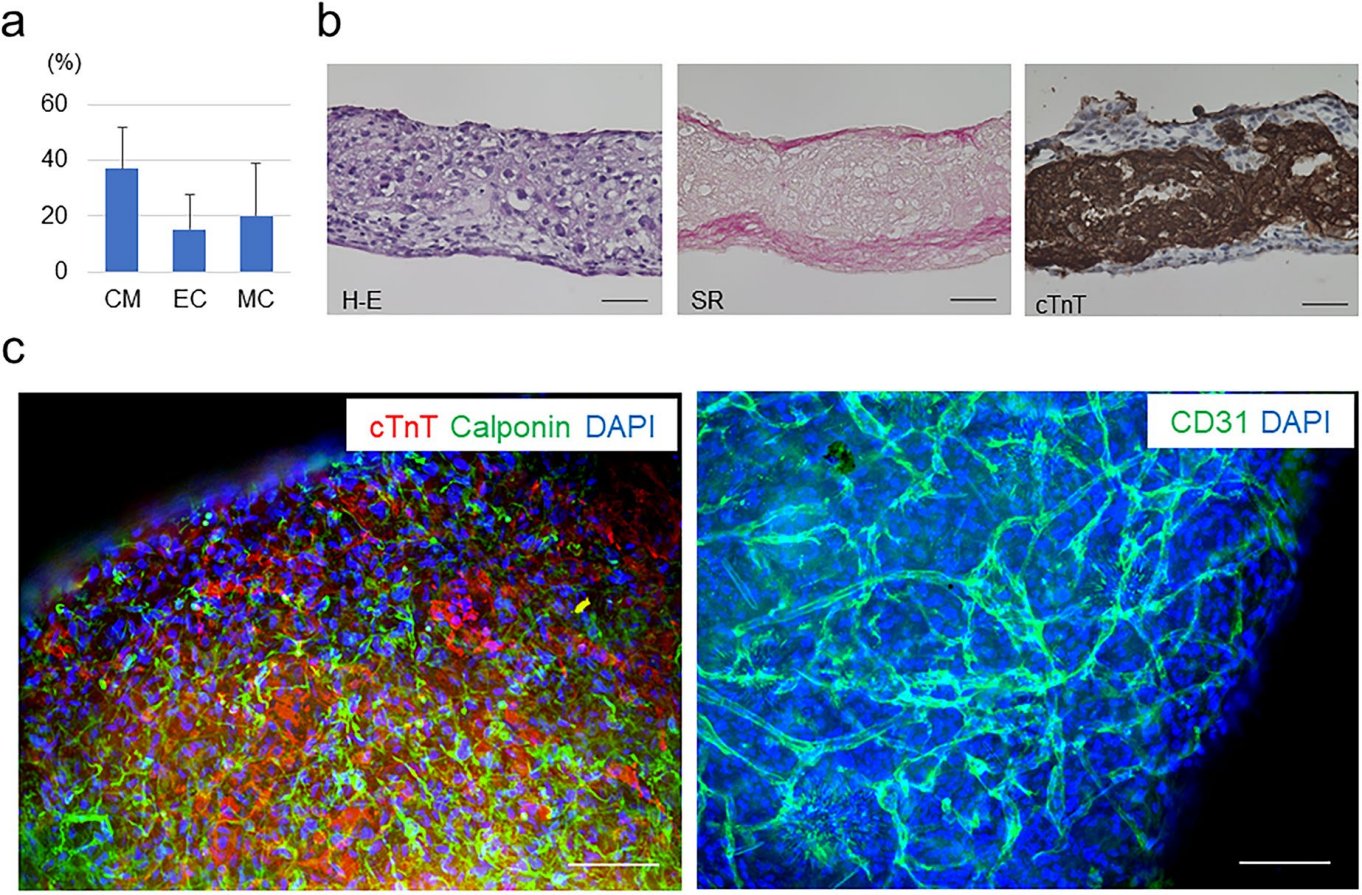
Preparation of 3D cardiac microtissues. (**a**) Cellular components of prepared human iPSC-derived generated cardiac tissue sheets (n = 13). CM, cardiomyocytes; EC, vascular endothelial cells; MC, vascular mural cells. (**b**) Representative histological evaluations of 3D cardiac microtissues. Left: haematoxylin–eosin (H-E) staining; Middle: Sirius red (SR) staining; Right: cardiac isoform of troponin-T (cTnT) immunostaining. Scale bars = 50 µm. (**c**) Representative fluorescent immunostaining of 3D cardiac microtissues. Left: Double staining of cTnT (CMs) and calponin (MCs). Right: Staining of CD31 (ECs). DAPI, 4′,6-diamidino-2-phenylindole. Scale bars = 100 µm. In (**b**) and (**c**), Biorevo BZ-9000 (https://www.keyence.com/products/microscope/fluorescence-microscope/bz-9000/) (Keyence) was used.

First, we induced various types of cardiovascular cell lineages as previously reported, with modifications^24–26^. The induced multiple cardiovascular cells were seeded on temperature-responsive culture plates to harvest cell sheet-shaped human iPSC-derived cardiac microtissues^24^. The cardiac microtissues comprised 37.0 ± 14.6% of cardiac isoform of troponin-T (cTnT)-positive CMs, 15.4 ± 12.5% of vascular endothelial cadherin (VE-cadherin)-positive vascular endothelial cells (ECs) and 20.0 ± 19.0% of platelet-derived growth factor receptor-beta (PDGFRβ)-positive vascular mural cells (MCs) (n = 13) (Fig. 1a). Dynamic rocking culture has been reported to drive CM hypertrophy and to promote structural organization and maturation of the sarcoplasmic reticulum in human pluripotent stem cell-derived artificial cardiac tissues^27,28^. We utilized a dynamic culture to thicken cardiac microtissues for optimization for the organ-on-a-chip system. Histological analyses revealed that the 3D cardiac microtissues were composed of approximately 15 layers of cells with an approximately 100 µm-thick cTnT-positive CM layer (150 µm thickness in the whole tissue) and formed multilayered extracellular matrices that may support tissue stiffness (Fig. 1b). Fluorescent immunostaining of the 3D cardiac microtissues indicated that the microtissues were composed of cTnT-positive CMs and vascular cells that were distributed among the CMs. CD31-positive vascular endothelial cells formed an organized vascular network among the 3D cardiac microtissues (Fig. 1c).

### Preparation of HMDs

**Figure 2:**
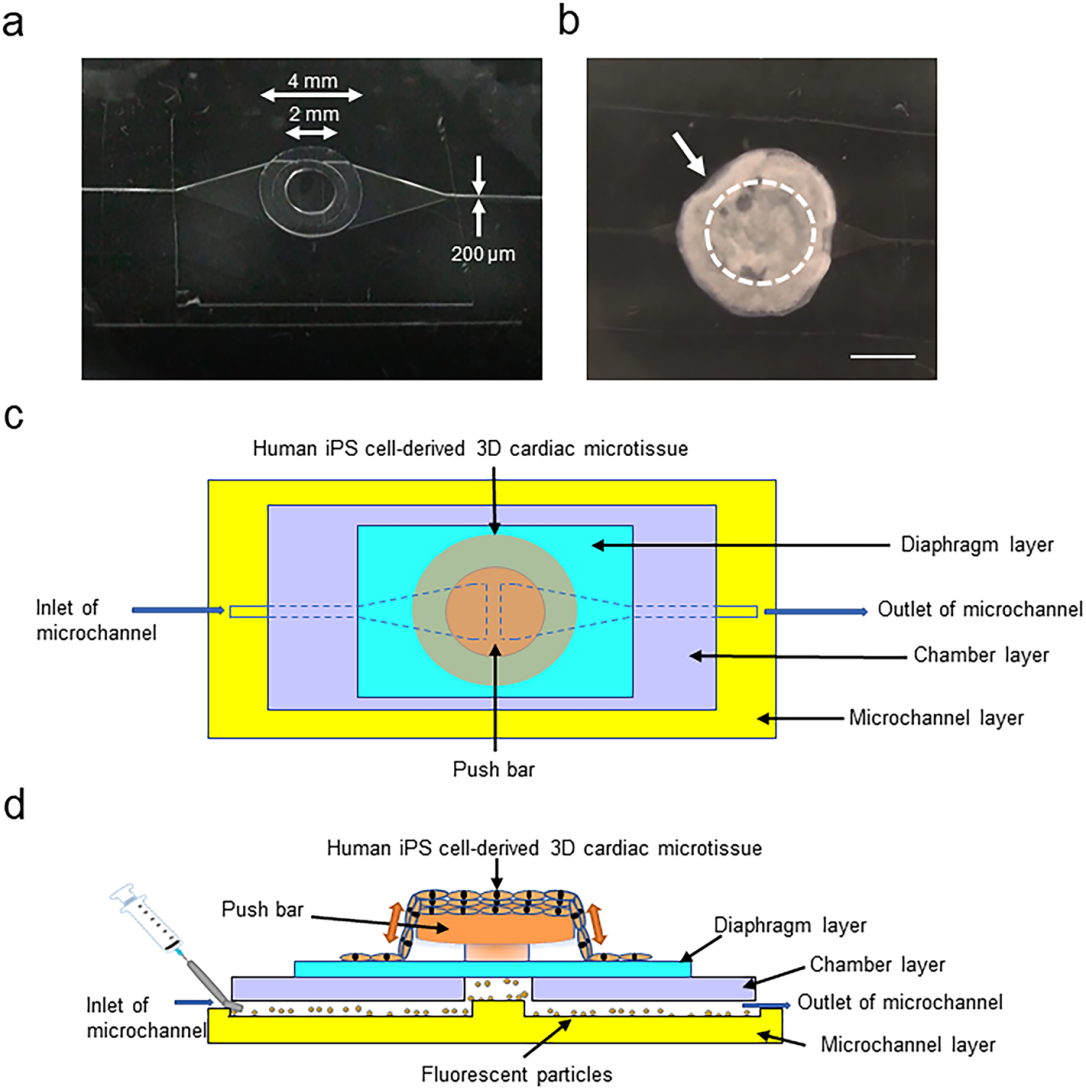
Preparation of HMDs. (**a**) Representative macroscopic view of the microfluidic chip. (**b**) Representative macroscopic view of the HMD. The arrow indicates the human iPSC-derived 3D cardiac microtissues attached to the microfluidic chip. The dotted circle indicates the position of the push bar. Scale bar = 2 mm. (**c**) (**d**) Schematic of the structure and working machinery of the HMD. (**c**) Top view. (**d**) Side view.

The detailed fabrication process of a microfluidic chip was described in a previous report^21^, and we provide a brief description here (Supplementary Fig. 1). The PDMS microfluidic chip (without check valves) consisted of four components: the microchannel, chamber, diaphragm, and push bar. The microchannels were fabricated using a replica molding method and a silicon wafer. PDMS prepolymer (Silpot 184 W/C, Dow Corning Toray, Tokyo, Japan) and photoresist (SU-8 3050, Nihon Kayaku, Tokyo, Japan) were used for this procedure. The depth and width of the microchannels were approximately 200 µm (Supplementary Fig. 1b). Other components were fabricated using 1-mm thick flat sheets of PDMS. The push bar was fabricated by cutting 2- and 4-mm diameter cylinders out of the PDMS sheet using a biopsy punch and stacking the 2 cylinders. The push bar was assembled on a 100-µm-thick diaphragm. The chamber was fabricated by cutting a 3-mm diameter circle in a 100-µm-thick PDMS sheet. The microchip components were aligned and stacked into the desired spatial configuration. All components were bonded with vacuum oxygen plasma^29^ at an intensity of 15 W and an oxygen flow rate of 5 mL/min for 0.5 min in the chamber of a compact etcher (FA-1, SAMCO, Kyoto, Japan) (Fig. 2a).

We sterilized and coated the microfluidic chip with 50 µg/mL fibronectin from bovine serum (Sigma, St. Louis, MO, USA) in phosphate-buffered saline (PBS) at 37 °C overnight to promote the attachment of 3D cardiac microtissues. After coating, we manually transferred the 3D cardiac microtissues onto the microfluidic chip and stabilized them for 2 h without medium, and then we added medium to prepare the heart-on-a-chip microdevice (HMD) (Fig. 2b). On the following day, we confirmed the spontaneous beating of the 3D cardiac microtissues, which were fairly well-attached to the push bar (at the centre of the microtissue) and diaphragm layer (at the periphery of the microtissue) of the microfluidic chip (activated HMD; Supplementary Video 1). The basic structure and working machinery of the HMD are illustrated in Fig. 2c,d.

### Validation of the HMD as a system for the assessment of tissue function

**Figure 3:**
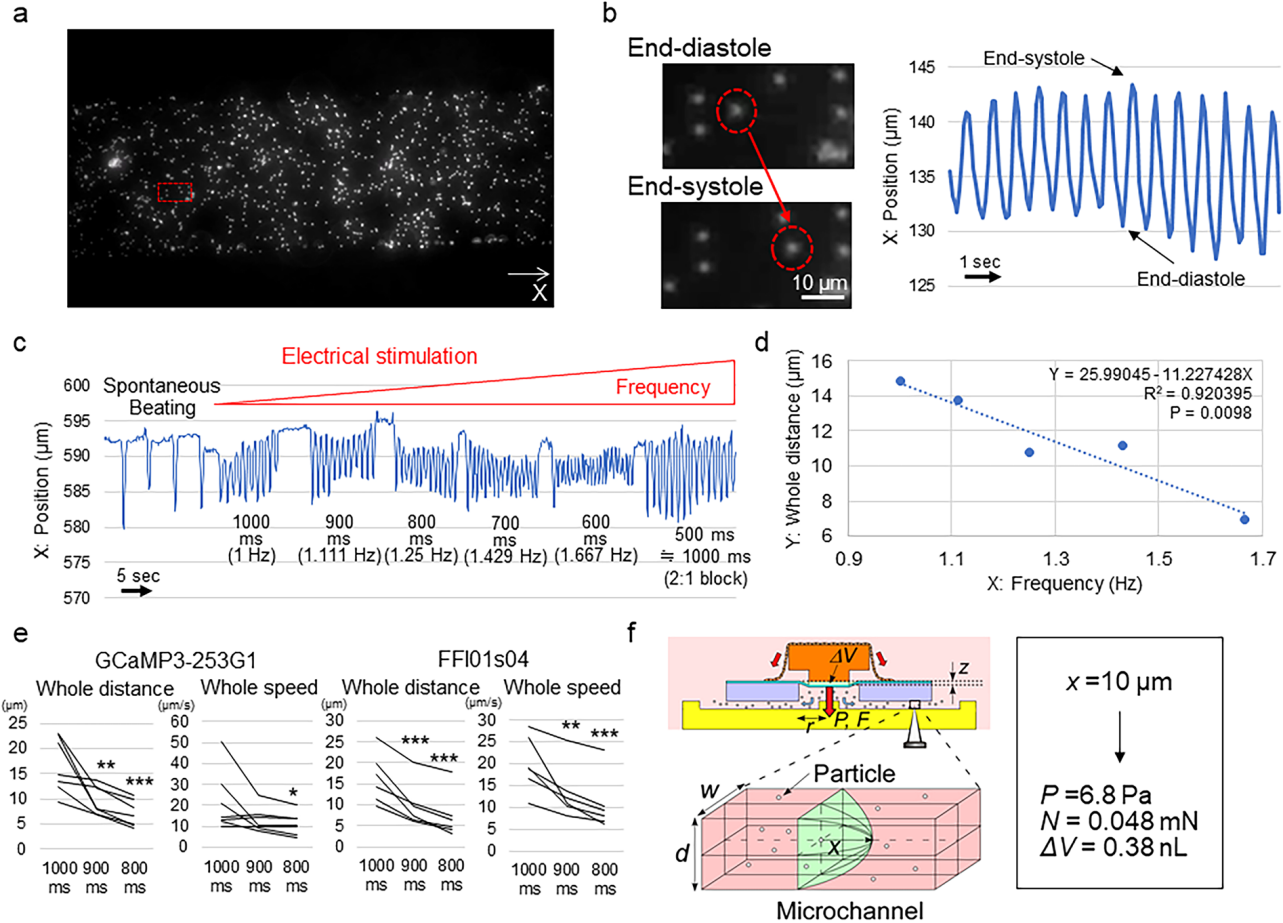
Detection of the particle displacement of the HMD and the analysis of tissue function. (**a**) Representative observation of microchannels loaded with particles. X indicates the axis of the particle position. The red square indicates the selected location in (b). (**b**) Representative pattern of particle displacement. The red dotted circles indicate the same particle at the end-diastolic (top) and end-systolic (bottom) phases during the pulsation of 3D cardiac microtissues. (**c**) Representative changes in the particle position in accordance with the interval and frequency of the electrical stimulation. After 500-ms interval stimulation, the HMD could capture 1 time point in 2 stimulations (2:1 block), which is equivalent to 1000-ms interval stimulation. (**d**) Representative relationship between the frequency of electrical stimulation (X) and the particle displacement distance in the whole beating cycle (Y). The results of the regression analysis are shown. All data are shown in Supplementary Fig. 2a. (**e**) Particle displacement distance and speed of the whole beating cycle at each electrical stimulation interval in HMDs constructed from the GCaMP3-253G1 (n = 7) and FFI01 s04 (n = 6) human iPSC lines, respectively. *P < 0.05. **P < 0.01. ***P < 0.001. (**f**) Calculation of the physiological parameters. *ΔV* = stroke volume, *z* = vertical displacement of the diaphragm, *P* = applied pressure, *F* = applied force, *r* = radius of the chamber, *w* = width of the microchannel, *d* = depth of the microchannel, and *x* = particle displacement distance. In (**a**) and (**b**), cellSens Standard (version 1.18) (https://www.olympus-lifescience.com/en/software/cellsens/) (Olympus) was used.

We loaded fluorescent particles (Fluoresbrite Plain Microspheres, 2.5% Solids-Latex, 2.0 μm YG, Polysciences, Inc., Warrington, FL, USA) into the microchannel. We successfully observed simultaneous particle displacement in accordance with the timing of the beating of the 3D cardiac microtissues, proving the basic concept of the HMDs (Fig. 3a,b; Supplementary Video 2). We electrically stimulated the HMDs with various frequencies and found that the particle displacement distance and speed were strongly correlated with the frequency of electrical stimulation^30^ (Fig. 3c,d; Supplementary Fig. 2a). The R^2^ values in the regression analyses of the HMDs generated from 2 different human iPS cell lines were 0.89 ± 0.08 (n = 7) for the GCaMP330-253G1 line and 0.92 ± 0.04 (n = 6) for the FFI01s04 line. The distance and speed of particle displacement in the systolic phase, diastolic phase and whole beating cycle were significantly decreased according to the increase in the frequency of the electrical stimulation (Fig. 3e; Supplementary Fig. 2b,c). These results indicate that the HMD represents pump function of 3D cardiac microtissues.

We calculated the stroke volume from the following equation based on the assumption that the microfluidic chip was symmetrical^31^ (Fig. 3f):

1 $$ \Delta V = 0.{94}wdx $$

where Δ*V* is stroke volume and *w* and *d* are the width and depth of the microchannel, respectively. Additionally, the displacement of the diaphragm was roughly calculated by the following equation:

2 $$ \Delta V = \left( {{1}/{3}} \right)\pi r^{{2}} z $$

where *r* is the radius of the chamber and *z* is the vertical displacement of the diaphragm. In cases of a circular diaphragm without initial stress, the vertical applied pressure on the diaphragm can be roughly calculated from the following equation:

3 $$ P = (C_{{1}} \sigma t/r^{{2}} )z + (C_{{2}} Et/r^{{4}} )z^{{3}} $$

where *P* is the applied pressure, *C*_*1*_ and *C*_*2*_ are constants depending on the diaphragm geometry and material, σ is the residual stress of the PDMS, *E* is Young’s modulus for the PDMS, and *t* is the diaphragm thickness.

The force applied on the membrane can be calculated by the following equation:

4 $$ F = \pi r^{2} P $$

In the present experiments, *w* = *d* = 200 μm, *r* = 1.5 mm and *t* = 100 μm based on the designed parameters. In the case of the PDMS circular diaphragm, *C*_1_ = 4, *C*_2_ = 3.5, σ = 240 kPa, and *E* = 750 kPa^32^. When *z* is small (smaller than 10 μm), the term in the second section of Eq. (3) can be neglected, and then *F* and *P* are proportional to *x* from Eqs. (1–4). For example, when *x* = 10 μm, *P* and *F* are calculated as 6.8 Pa and 0.048 mN, respectively. The systolic volume (*ΔV*) was also calculated as 0.38 nL (= 0.*94 wdx*). In the present experiments, the detected systolic displacement distance was 8.3 ± 2.7 μm (4.9 – 13.0 μm) (calculated as 0.040 ± 0.013 mN; 0.024 – 0.063 mN) at 1000-ms intervals, which decreased to 3.6 ± 2.0 μm (1.6 – 5.1 μm) (calculated as 0.017 ± 0.010 mN; 0.0077 – 0.025 mN) at 800-ms intervals, indicating that the HMD system can detect a substantially small force of < 0.01 mN.

### MUSCLEMOTION analysis

**Figure 4:**
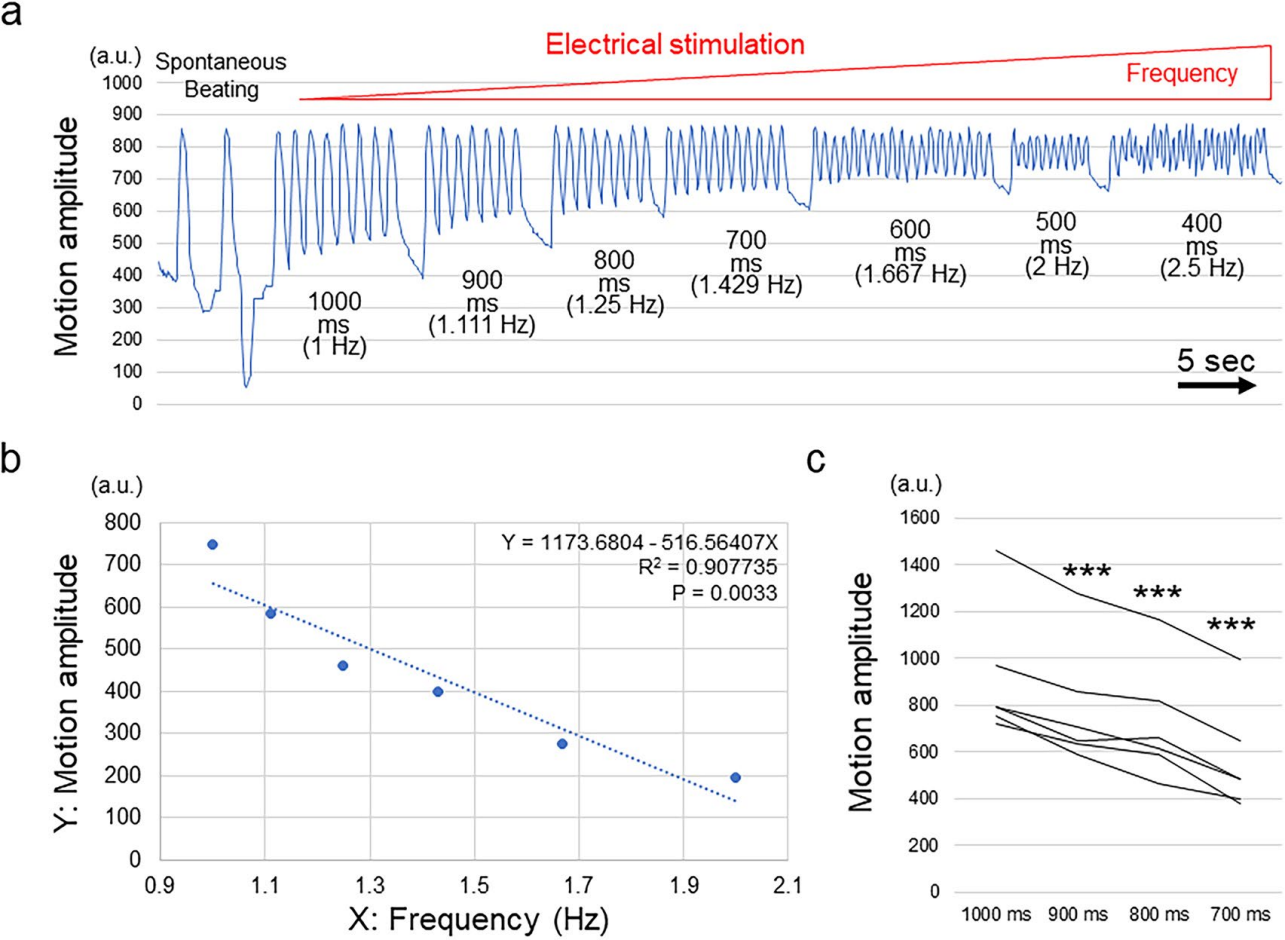
MUSCLEMOTION analysis of 3D cardiac microtissues. (**a**) Representative changes in the motion amplitude in accordance with the interval and frequency of electrical stimulation. (**b**) Representative relationship between the frequency of electrical stimulation (X) and the motion amplitude in the whole beating cycle (Y). The results of the regression analysis are shown. All data are shown in Supplementary Fig. 3. (**c**) Motion amplitude of the whole beating cycle at each electrical stimulation interval in 3D cardiac microtissues (n = 6). ***P < 0.001.

To validate the capacity of HMDs to evaluate contractile function, we tested the 3D cardiac microtissues with another already reported video-based system to evaluate contractile function. We used MUSCLEMOTION, a versatile open-source software, to quantify CM and cardiac muscle contraction^33^. Along with the increased frequency of electrical stimulation, the calculated motion amplitudes of the whole beating cycle decreased and showed a strong correlation with the stimulation frequency similar to that observed in the HMD experiments (R^2^ = 0.96 ± 0.03; n = 6) (Fig. 4a,b; Supplementary Fig. 3). The motion amplitudes in the whole beating cycle significantly decreased according to the increase in the frequencies of electrical stimulation (Fig. 4c). These results indicate that the contractile function of cardiac muscle tissues evaluated by using the HMDs was relevant to a previously reported method used to evaluate contractile function.

### Transition of the intracellular calcium ion concentration of CMs

**Figure 5:**
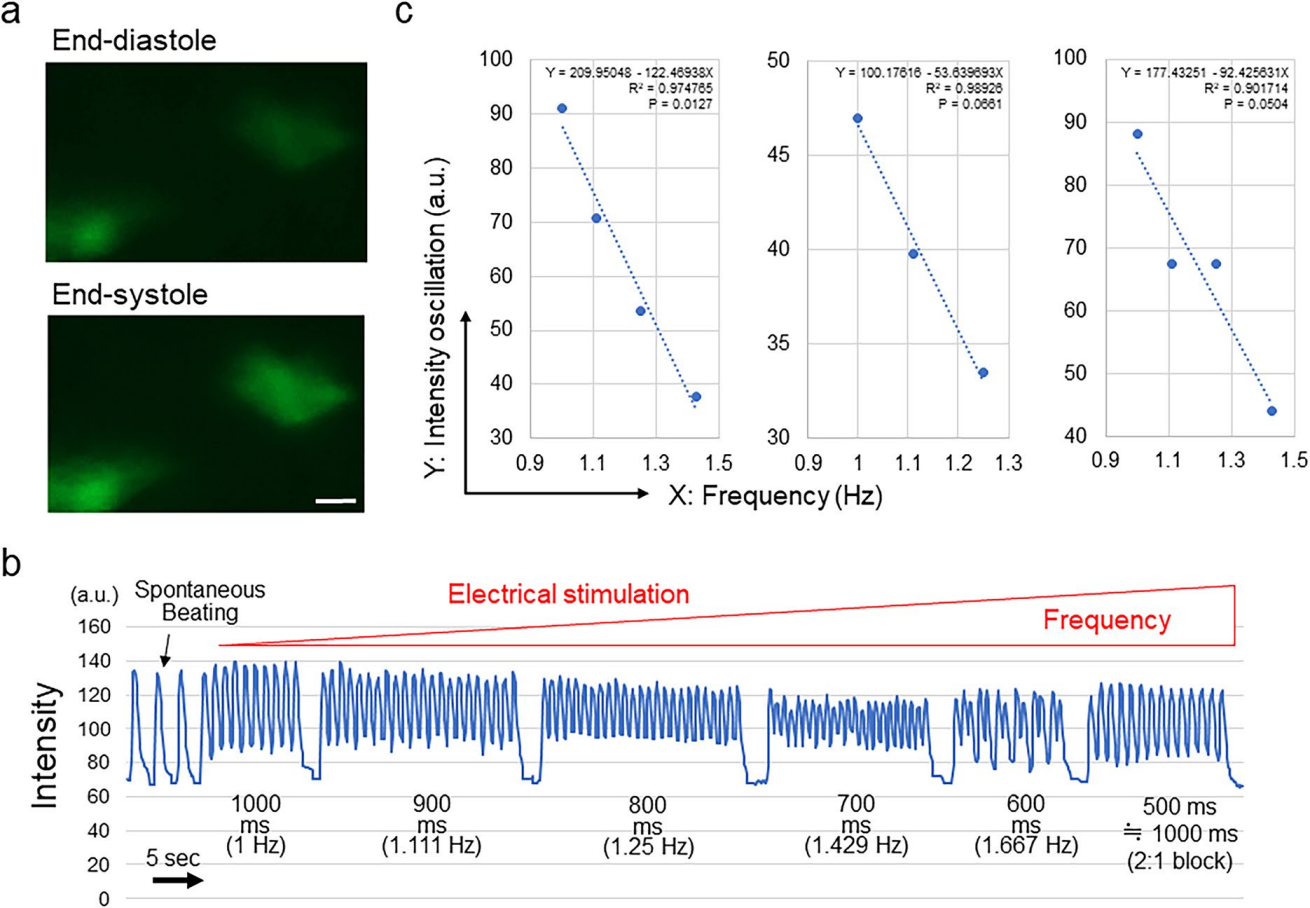
Intracellular calcium oscillation analysis of 3D cardiac microtissues. (**a**) Representative visualization of human iPSC (GCaMP3-253G1)-derived CMs at end-diastole (top) and end-systole (bottom). Scale bar = 10 µm. (**b**) Representative changes in the signal intensity in accordance with the interval and frequency of electrical stimulation. Upon 500-ms interval stimulation, 3D cardiac microtissues could capture 1 time point in 2 stimulations (2:1 block), which is equivalent to 1000-ms interval stimulation. (**c**) Relationship between the frequency of electrical stimulation (X) and the oscillation of the signal intensity in the whole beating cycle (Y). The results of the regression analysis are shown. In (**a**), cellSens Standard (version 1.18) (https://www.olympus-lifescience.com/en/software/cellsens/) (Olympus) was used.

Next, we evaluated the transition of the intracellular calcium ion concentration of CMs composing 3D cardiac microtissues in accordance with electrical stimulation using the GCaMP3-253G1 iPSC line expressing GCaMP3, a genetically encoded calcium sensor^34,35^ (Fig. 5a; Supplementary Video 3). As expected, the oscillation of the concentration of calcium ions indicated by the GCaMP3 signal intensity showed a strong negative correlation with the beating frequency (R^2^ = 0.96 ± 0.05; n = 3) (Fig. 5b,c). These results indicate that the contractile function of cardiac muscle tissues evaluated by using HMDs was correlated with the transition of the intracellular calcium ion concentration of CMs, which is a fundamental biological component of CM contraction.

### Drug test

**Figure 6:**
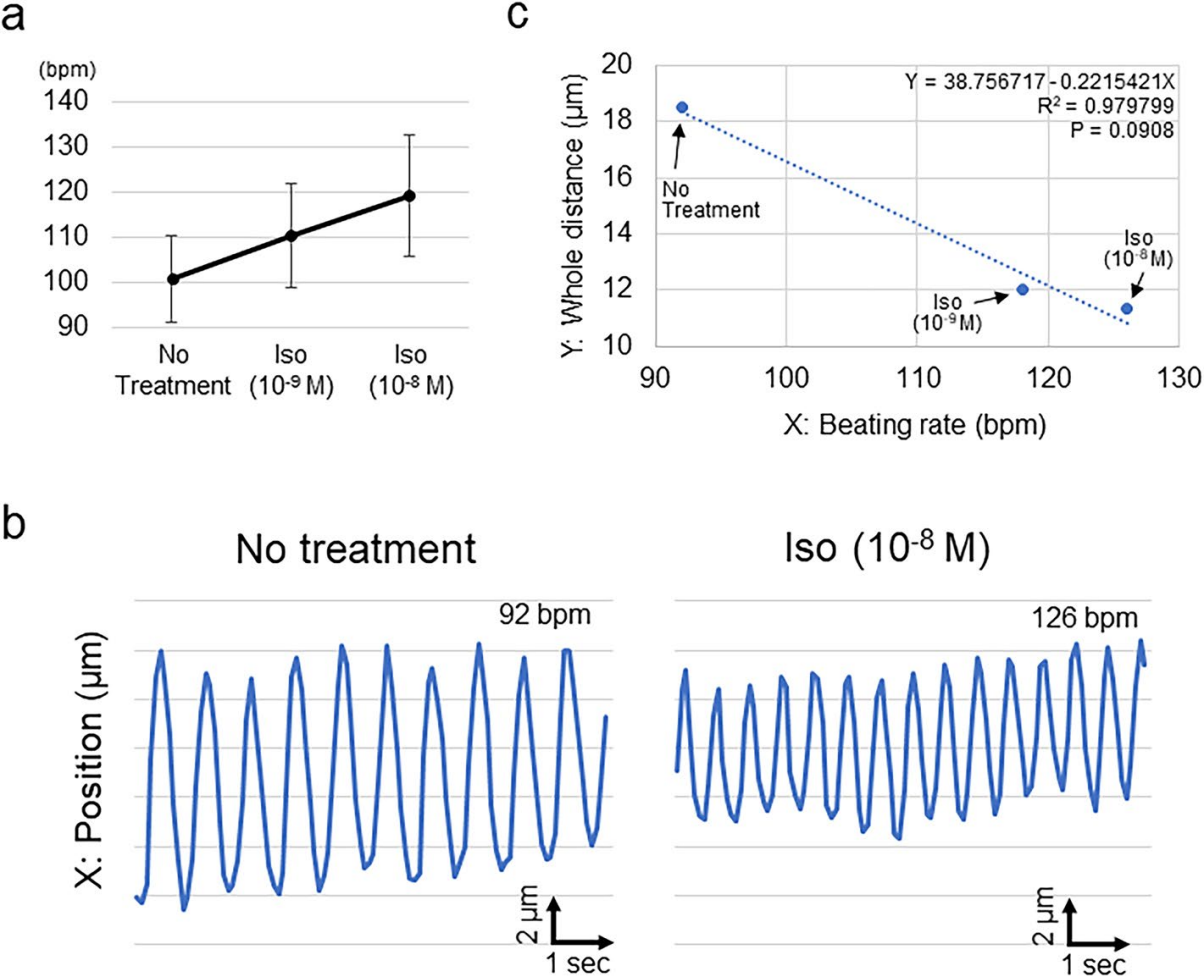
Pharmacological responses of HMDs to isoproterenol. (**a**) Dose-dependent change in the beating rate of HMDs. Bpm, beats per minute; Iso, isoproterenol. (**b**) Representative changes in the particle position in accordance with the administration of isoproterenol. Left: no treatment; Right, administration of isoproterenol (10^–8^ M). (**c**) Representative relationship between the beating rate of the HMD (X) and the particle displacement distance in the whole beating cycle (Y). The results of the regression analysis are shown. All data are shown in Supplementary Fig. 4.

Finally, we treated the HMD with isoproterenol, a representative β-adrenoceptor agonist. The beating rate of the 3D cardiac microtissues mounted on the microfluidic chip increased dose-dependently (Fig. 6a). We confirmed that the entire particle displacement distance correlated with the beating rate, which changed in accordance with the isoproterenol treatment dose (R^2^ = 0.87 ± 0.17; n = 4) (Fig. 6b,c; Supplementary Fig. 4), indicating that the HMD could accurately depict pharmacological responses to an inotropic reagent.

## Discussion

In the present study, we reported the HMD, a new bioassay system based on human iPSC technology and MEMS, that can be utilized for drug discovery and cardiac toxicity tests to evaluate physiological heart function. The HMD showed a significant correlation between particle displacement and the beating frequency of the human iPSC-derived 3D cardiac microtissues between 1 and 2 Hz, which is relevant to the physiological condition of the human heart rate at rest and allowed us to evaluate the force-frequency relationship (FFR), a measure of tissue function and maturation^30,36^. The correlation was also validated by examination of the calcium oscillation patterns of CMs according to the pulsation of 3D cardiac microtissues and video-based motion analysis, in which the FFRs showed transition patterns similar to those of the HMD. The HMD could react with a representative β-adrenergic agonist to produce beating frequency-dependent changes in particle displacement.

Medical applications of HMDs include exploratory research on drug discovery for cardiovascular diseases and drug safety tests through in vitro tests on human tissue surrogates, to avoid preclinical animal studies before market release^17^. HMDs may hold promise as a tool for “order-made” cardiac toxicity tests, since the HMDs can be prepared by patient-derived iPSCs requiring specific medications, such as anticancer drugs, for which the cardiac toxicity varies according to the biological backgrounds of the individuals. Furthermore, HMDs created with heart disease-specific human iPSCs can be used for the discovery of disease-specific treatment drugs through high-throughput screening of chemical compound libraries^37–39^. In addition to the validation of the basic system of the HMD, as shown in the present study, further medical applications of the HMDs as described above should be investigated in our next work, in which further optimization of the HMD system and experimental conditions such as the medium/buffer with defined chemical factors during drug tests, manipulations to firmly attach the 3D cardiac microtissue onto the microfluidic chip, and the identification of factors that might affect the tissue function and variation of data (e.g., cellular composition, 3D structure, and cellular alignments) should be conducted.

There have been few reports that evaluated the small contractile force of human iPSC-derived cell sheet-shaped cardiac tissues so far. Sasaki et al. recently reported a force measurement system designed for human iPSC-derived cardiac cell sheet tissues involving the attachment of cardiac cell sheets to fibrin gel sheets and mounting them to a force transducer to directly measure the contractile force^20^. In the present study, we succeeded in evaluating the physiological parameters of the cardiac tissues, including the developing pressure and fluidic output, which are parameters that function as surrogates for the actual pump function and may provide a new concept in this research field. Although we could not generate a Frank-Starling curve^40^ by experimentally measured values because of technical difficulties in measuring the developing pressure in small microchannels, we could generate a Frank-Starling curve by a calculation using our HMD system (Supplemental Fig. 5). We confirmed a linear relationship between applied pressure to the diaphragm (near to the fluid pressure in low flow rate condition of this estimation) and systolic volume in the value range we have shown in Fig. 3c (Supplementary Fig. 5a). However, the curve deviated from the linear relationship in accordance with the increase of systolic volume and applied pressure in the calculation (Supplementary Fig. 5b) which would be a similar pattern with Frank-Starling curves in heart tissues. The results of the calculated Frank-Starling curve might indicate a future possibility of the establishment of a Frank-Starling curve based on measured values using the HMD system, and the experimental validation of the relationship between the pressure and systolic volume to recapitulate human heart physiology would further promote the clinical implementation of the present system. Our fluidic channel and membrane-based system, which can be prepared with relatively low costs without a special apparatus, would be advantageous for a broad use in medical research fields in the future.

Furthermore, the present system has the advantage of showing a wider dynamic range in the measurement of the forces of cell sheets. Even though the forces of cell sheets have been measured by cantilever- and fibrin gel sheet-based systems thus far^18–20^, the reported systems could not measure a small force under 0.1 mN due to bulk scale detection. On the other hand, the detection limit of the present study is approximately 0.0077 mN, which corresponds to the displacement of a particle displacement distance of 1.6 μm in a microchannel. This represents an improvement in the dynamic range of 2 orders of magnitude and is based on the use of a microfluidic device as the machinery to detect and quantify the force. A recent study reported a measurement system for the pressure produced by the cell sheet contractile force utilizing a tube structure prepared by cell sheets^41^. The detection limit of the tube-based method is approximately 4 Pa, which is larger than that of the present method, which is 1.1 Pa and corresponds to the displacement of a particle by 1.6 μm in a microchannel. Importantly, the sensitivity can be further improved as well when smaller microchannels are prepared. Even compared with our previous CM-based pump systems^21,42,43^, the present system shows advantages regarding stability and reproducibility by virtue of the use of human iPSC-derived thick 3D cardiac microtissues produced by dynamic rocking culture of cell sheets, which was first developed and reported in the present study and enabled us to stably conduct quantitative evaluations of tissue function.

Contraction of CMs is strongly related to the transition of the intracellular calcium ion concentration, which is a fundamental biological process of CMs occurring during contraction and relaxation involving the coupling of actin and myosin filaments^44^. In the present study, we confirmed that particle displacement according to the frequency of external electrical stimulation evaluated by the HMD changes in a way similar to the intracellular concentration of calcium ions, which was visualized by the intensity of the GCaMP signals^34^. This result indicates that the HMD could successfully demonstrate the correlation between intracellular calcium ion turnover and the contractile force, which underlies the biological machinery of CM contraction.

The immaturity of human iPSC-derived CMs in terms of potassium ion channels and the functioning of the sarcoplasmic reticulum in intracellular calcium ion turnover is reported as a drawback for the clinical application of human iPSCs in regenerative medicine and drug discovery^45,46^. In our preliminary studies, in which we exposed human iPSC-derived cardiac microtissues to electrical stimulation just after preparation, we could not sufficiently observe stimuli-dependent pulsations (data not shown), possibly indicating the functional immaturity of the microtissues. To overcome this, we attempted dynamic rocking culture with cyclic see-sawing stimuli using digital rockers, which are reported to promote the tissue maturation of human pluripotent stem cell-engineered cardiac tissues^27,28^. Although we could not confirm a positive FFR in the present experiments, which is a characteristic of mature myocardial tissues^30,36^, the dynamic culture led to the formation of a thick myocardium, which was confirmed by histological evaluations, and our revised system with the dynamic culture allowed us to stably control the pulsation frequency in response to electrical and pharmacological stimuli and to sufficiently evaluate the particle displacement in the presence of changes in the pulsation frequency of the thick 3D cardiac microtissues. Utilization of previously reported strategies to promote human pluripotent stem cell-derived CM maturation, such as the administration of thyroid and glucocorticoid hormones or fatty acids during culture^47–49^, would further promote tissue maturation and consequently change the intrinsic contractile properties of cardiac microtissues, which should be attempted in our next work.

In conclusion, we have developed the HMD as a sensitive bioassay system to detect small changes in the physiological parameters of human iPSC-derived 3D cardiac microtissues as a surrogate for human heart tissue function, which can be used for investigations for drug discovery and cardiac toxicity tests. Further validation of the system with candidate drugs and chemicals would be anticipated to confirm the usefulness of the HMD in terms of its pharmaceutical application.

## Methods

All methods were carried out in accordance with the relevant guidelines and regulations (Declaration of Helsinki).

### Maintenance of human iPS cells and differentiation of cardiovascular cell lines

Two different human iPSC lines were used in the present study: the GCaMP3-253G1 line expressing GCaMP3, a genetically encoded calcium sensor^34,35^, and the FFI01 s04 line^50^, which is a human leukocyte antigen homozygous iPSC line established at the Center for iPS Cell Research and Application (CiRA), Kyoto University, Kyoto, Japan, and distributed subject to the informed consent of a healthy donor and the permission of the Institutional Review Board of RIKEN and CiRA. A written informed consent was obtained from the patients to establish the iPSC line at CiRA. All experimental protocols were approved by the Institutional Review Board of RIKEN.

The maintenance of human iPSCs and differentiation of cardiovascular cells were conducted in accordance with our previous studies^24–26^ with modifications. In brief, iPSCs were expanded and maintained with StemFit AK02N medium (AJINOMOTO, Tokyo, Japan). At confluence, the cells were dissociated with TrypLE Select (Thermo Fisher Scientific, Waltham, MA, USA), dissolved in 0.5 mM ethylenediaminetetraacetic acid in PBS (1:1) and passaged as single cells (5,000 – 8,000 cells/cm^2^) every 5–7 days in AK02N containing iMatrix-511 silk (FUJIFILM Wako Pure Chemical Corp., Osaka, Japan) (0.125 µg/cm^2^) (uncoated laminin fragment)^51^ and ROCK inhibitor (Y-27632, 10 µM, FUJIFILM Wako). Penicillin–streptomycin (Thermo Fisher Scientific) (10,000 U/mL) (1:100 dilution) was used as required. For cardiovascular cell differentiation, single iPSCs were seeded onto Matrigel-coated plates (1:60 dilution) at a density of 300,000–400,000 cells/cm^2^ in AK02N with Y-27632 (10 µM). At confluence, the cells were covered with Matrigel (1:60 dilution in AK02N) one day before induction. We replaced the AK02N medium with RPMI + B27 medium (RPMI 1640, Thermo Fisher; 2 mM L-glutamine, Thermo Fisher; 1× B27 supplement without insulin, Thermo Fisher) supplemented with 100 ng/mL Activin A (R&D, Minneapolis, MN, USA) (differentiation day 0; d0) [3–5 µM CHIR99021 (Tocris Bioscience, Bristol, UK) was added as required] for 24 h, which was followed with supplementation with 10 ng/mL bone morphogenetic protein 4 (BMP4; R&D) and 10 ng/mL basic fibroblast growth factor (bFGF) (d1) for 4 days without a culture medium change. At d5, the culture medium was replaced with RPMI1640 medium supplemented with 50 ng/ml vascular endothelial cell growth factor (VEGF)_165_ (FUJIFILM Wako) [2.5 µM IWP4 (Stemgent, Cambridge, MA, USA) and 5 µM XAV939 (Merck, Kenilworth, NJ, USA) were added as required]. The culture medium was refreshed with RPMI1640 supplemented with 50 ng/ml VEGF every other day. Beating cells appeared at d11 to d15. To control the percentages of MCs sufficient to form cell sheets, we used a part of differentiation culture for MC differentiation^25^ and induced the differentiation of MCs as required; after d3, the culture medium was replaced with RPMI + FBS medium [RPMI1640, 2 mM of L-glutamine, 10% foetal bovine serum (FBS)] and was refreshed every other day.

### Generation and maturation of cardiac microtissues

After differentiation (d13–15), cells were dissociated by incubation with Accumax (Innovative Cell Technologies, San Diego, CA, USA), and the cell population was measured by flow cytometry of the mixture of cells. The cell mixture was plated onto an FBS-coated 12-well multiwell UpCell plate (CellSeed, Tokyo, Japan) at 1,500,000 – 4,000,000 cells/well with 2 mL of attachment medium [AM; alpha minimum essential medium (αMEM) (Thermo Fisher) supplemented with 10% FBS and 5 × 10^−5^ M of 2-mercaptoethanol] containing 25–50 ng/ml VEGF and 10 µM of Y-27632. After two days in culture, 25–50 ng/ml VEGF was added to the culture medium. After two more days in culture, the cells were moved to room temperature. Within 15–30 min, the cells detached and floated in the media as monolayer cardiac microtissues. The collected cardiac microtissues were allowed to reattach to Matrigel-coated dishes and incubated with AM containing 50 ng/ml Y-27632 for 24 h. For the maturation of cardiac microtissues, we used dynamic rocking culture as previously reported^27,28^. We cultured the reattached cardiac microtissues on a Compact Digital Rocker (Thermo Fisher, #88880019) at 60 rpm and 13 degrees for 9–14 days. The medium was refreshed with AM once every 2–3 days.

### Flow cytometry

Flow cytometry was conducted in accordance with our previous study with modifications^24^. Cardiac microtissues were dissociated by incubation with Accumax and stained with one or a combination of the following surface markers: anti-PDGFRβ conjugated with phycoerythrin (PE), clone 28d4, 1:100 (BD, Franklin Lakes, NJ, USA), and anti-VE-cadherin conjugated with allophycocyanin (APC), clone 55-7h1, 1:100 (BD). To eliminate dead cells, cells were stained with the LIVE/DEAD fixable Aqua dead cell staining kit (Thermo Fisher). For cell surface markers, staining was carried out in PBS with 5% FBS. For intracellular proteins, staining was carried out in cells fixed with 4% paraformaldehyde (PFA) in PBS. Cells were stained with the anti-cardiac isoform of troponin T (cTnT) (clone 13-11) (Thermo Fisher) labelled with APC using Zenon technology (Thermo Fisher) (1:50). The staining was performed in PBS with 5% FBS and 0.75% saponin (Sigma). The stained cells were analysed by a BD FACS Aria II (BD) or CytoFLEX S (Beckman Coulter, Brea, CA, USA). Data were collected from at least 10,000 events. Data were analysed with DIVA software (BD) or CytExpert software (Beckman Coulter).

### Histological analysis and fluorescence microscopy

Three-dimensional cardiac microtissues were fixed in 4% PFA and embedded in paraffin. Sections (6-μm thickness) were prepared and stained with haematoxylin–eosin and Sirius red. For fluorescence microscopy, 3D cardiac microtissues were stained for cTnT (Thermo Fisher) (1:500), calponin (anti-calponin1 antibody, clone EP798Y) (Abcam, Cambridge, UK) (1:500) and CD31 (monoclonal mouse IgG_1_, clone 9G11) (R&D) (1:50) with DAPI (4′,6-diamidino-2-phenylindole) (Thermo Fisher). Anti-mouse Alexa 546 (Thermo Fisher), anti-rabbit Alexa 488 (Thermo Fisher) and anti-mouse Alexa Fluor 488 (Thermo Fisher) were used as secondary antibodies. The tissues were photographed with an all-in-one fluorescence microscopic system, Biorevo BZ-9000 (Keyence, Osaka, Japan).

### Methods for the evaluation of particle displacement

We acquired videos of the particle displacement by fluorescence microscopy (CKX53, OLYMPUS, Tokyo, Japan) by using a fluorescein isothiocyanate (FITC) filter with a camera system (DP27, OLYMPUS) and software (cellSens, OLYMPUS). Graphical user interface (GUI) software has been developed to facilitate the precise tracking of the particles based on videos of iPSC-derived tissue. RGB serial images were obtained from the captured videos and then converted into 8-bit intensity. The image dataset was filtered by a top-hat morphological filter (size = 9, disk shape) followed by an intensity averaging filter with a size of 3 × 3. This eliminated much of the background noise from the images and hence paved the way for precise particle tracking. Our tracking strategy starts with the initialization step, in which the user is asked to click, roughly, on the particles that need to be tracked. Each clicked point is used by the software to estimate a region of interest (ROI) that covers the entire particle. It is assumed that the highest intensity value inside an ROI will represent the central x–y position of the particle. The same x–y position will be used to determine the ROI of the same particle in the following serial image. Again, the highest intensity value inside the ROI was detected, and a new central x–y position of the particle was determined. By repeating this procedure, we were able to automatically track the location of the particles over the entire inputted serial images. The x–y coordinates of the particle locations at each time point were recorded in an Excel file for further analysis. For each condition, the averages for the results of 4–5 consecutive waves are recorded and analysed.

### Electrical stimulation test

For electrical stimulation of HMDs or 3D cardiac microtissues, we used a custom-made electrical stimulation system with an electrical pulse generator (Strex, Osaka, Japan). We installed 2 platinum electrodes in parallel on 3D cardiac microtissues cultured in 6-well multiwell plates to conduct field electrical stimulation through the medium. We used electrical stimulation at a voltage of 30 V (peak-to-peak) with a 4 ms pulse width and a 1000 ms interval (1 Hz), which was gradually decreased to 500 ms (2 Hz). The capture of electrical stimulation on the 3D cardiac microtissues was confirmed by simultaneous microscopic observations.

### MUSCLEMOTION analysis

MUSCLEMOTION is a versatile open-source software with a video-based system used to evaluate contractile function^33^. We used the software as the provider instructed. In brief, we used ImageJ software^52^ and installed MUSCLEMOTION as a plug-in. The motion amplitude was used for analysis. For the preparation of the analysis, 3D cardiac microtissues were reattached onto fibronectin-coated culture plates (50 µg/mL, 37 °C, overnight) and subjected to video acquisition.

### Calcium imaging

The intracellular calcium ion concentration was visualized by measuring the GCaMP3 signals from 3D cardiac microtissues composed of the GCaMP3-253G1 iPSC line expressing GCaMP3, a genetically encoded calcium sensor^34,35^. Three-dimensional cardiac microtissues were allowed to reattach to fibronectin-coated culture plates (50 µg/mL, 37 °C, overnight) and subjected to analysis. We acquired videos of the signal intensity by fluorescence microscopy (CKX53) through a FITC filter with a camera system (DP27) and software (cellSens). Data analysis was conducted using ImageJ software^52^ with manually selected ROIs and the “Plot Z-axis Profile” function.

### Drug administration test

We dissolved (−)-isoproterenol hydrochloride (Iso) (Sigma) (#16504-100MG) in dimethyl sulfoxide to prepare a 1 M stock solution. We prepared 10^–9^ M and 10^–8^ M Iso solutions dissolved in AM by serial dilution. We serially changed the medium for the HMDs with AM without Iso (no treatment), AM with Iso (10^–9^ M) and AM with Iso (10^–8^ M). For data acquisition for each condition, we waited for 10 min at 37 °C and then subjected the HMDs to observation for particle displacement by microscopy (CKX53) and video acquisition. Several particles evenly distributed in the microchannel were selected and used for analysis, and the values were calculated as the average of the analysed particles. In the drug administration tests, the GCaMP3-253G1 cell line was used.

### Statistical analysis

Data are expressed as the mean ± SD. Statistical analysis was performed using one-way repeated measures analysis of variance or regression analysis.

## Supplementary information

Supplementary Information 1.

Supplementary Video 1.

Supplementary Video 2.

Supplementary Video 3.
