# Supplementary material for: Establishment of a heart-on-a-chip microdevice based on human iPS cells for the evaluation of human heart tissue function
Source: Sci Rep. 2020 Nov 5;10:19201. doi: 10.1038/s41598-020-76062-w (PMC7645446; doi:10.1038/s41598-020-76062-w)
Supplement: Supplementary file 1 — Supplementary Information 1. [file 41598_2020_76062_MOESM1_ESM.pdf]

Supporting Information

Establishment of a heart-on-a-chip microdevice based on human iPS cells for the evaluation of human heart tissue function

Mosha Abulaiti[1,2,3], Yaxiaer Yalikun[3], Kozue Murata[1,4], Asako Sato[3], Mustafa M. Sami[5], Yuko Sasaki[1], Yasue Fujiwara[1,2], Kenji Minatoya[2], Yuji Shiba[6], Yo Tanaka[3] and Hidetoshi Masumoto[1,2]

1: Clinical Translational Research Program, RIKEN Center for Biosystems Dynamics Research, Kobe, Japan

2: Department of Cardiovascular Surgery, Graduate School of Medicine, Kyoto University, Kyoto, Japan

3: Laboratory for Integrated Biodevice, RIKEN Center for Biosystems Dynamics Research, Suita, Japan

4: Institute for Advancement of Clinical and Translational Science, Kyoto University Hospital, Kyoto, Japan

5: Laboratory for Morphogenetic Signaling, RIKEN Center for Biosystems Dynamics Research, Kobe, Japan

6: Department of Regenerative Science and Medicine, Institute for Biomedical Sciences, Shinshu University, Matsumoto, Japan

Supplementary Figures

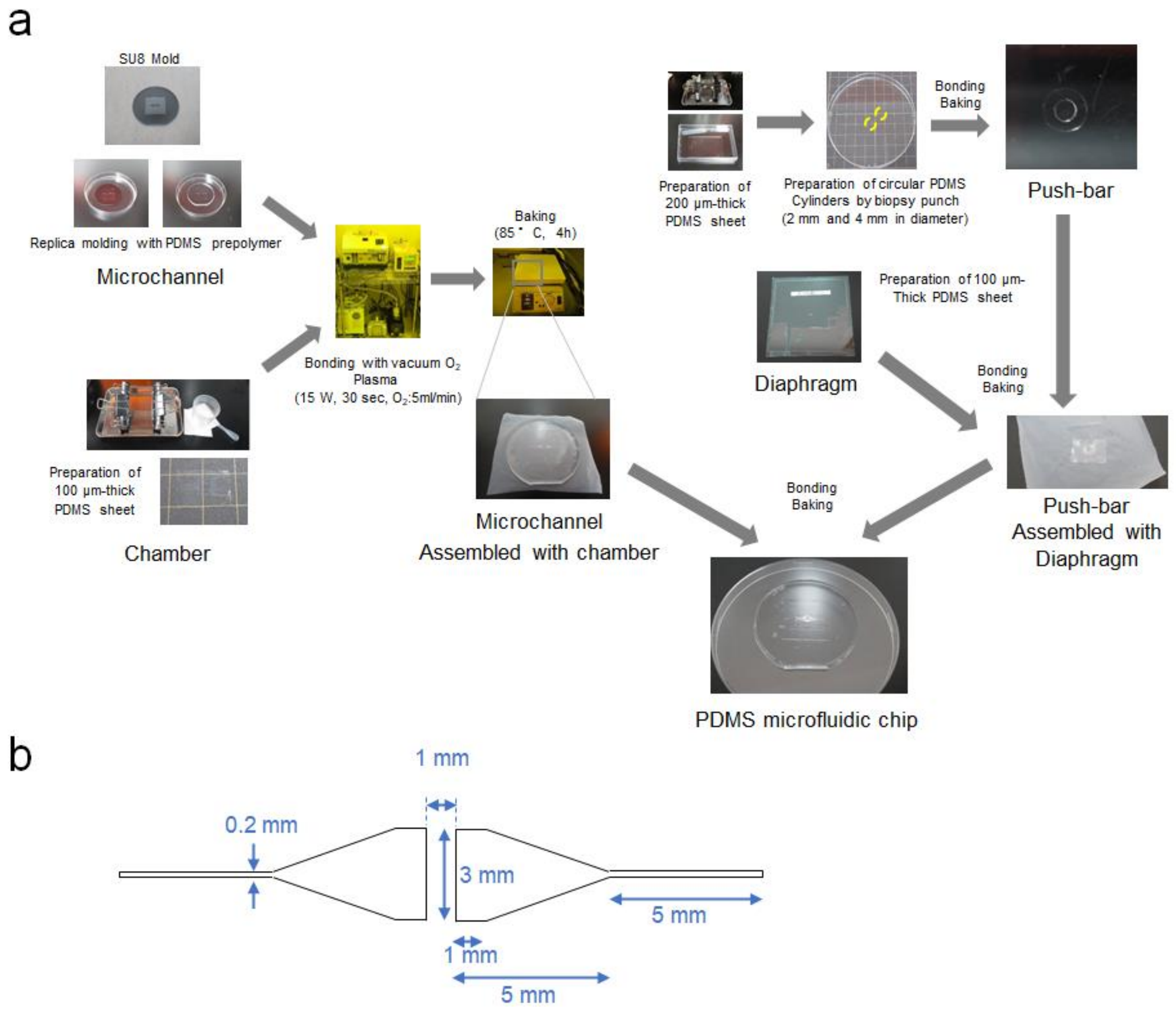

Supplementary Figure 1: Preparation of microfluidic chip.

(**a**) Fabrication processes of microfluidic chip. (**b**) Design of microchannel.

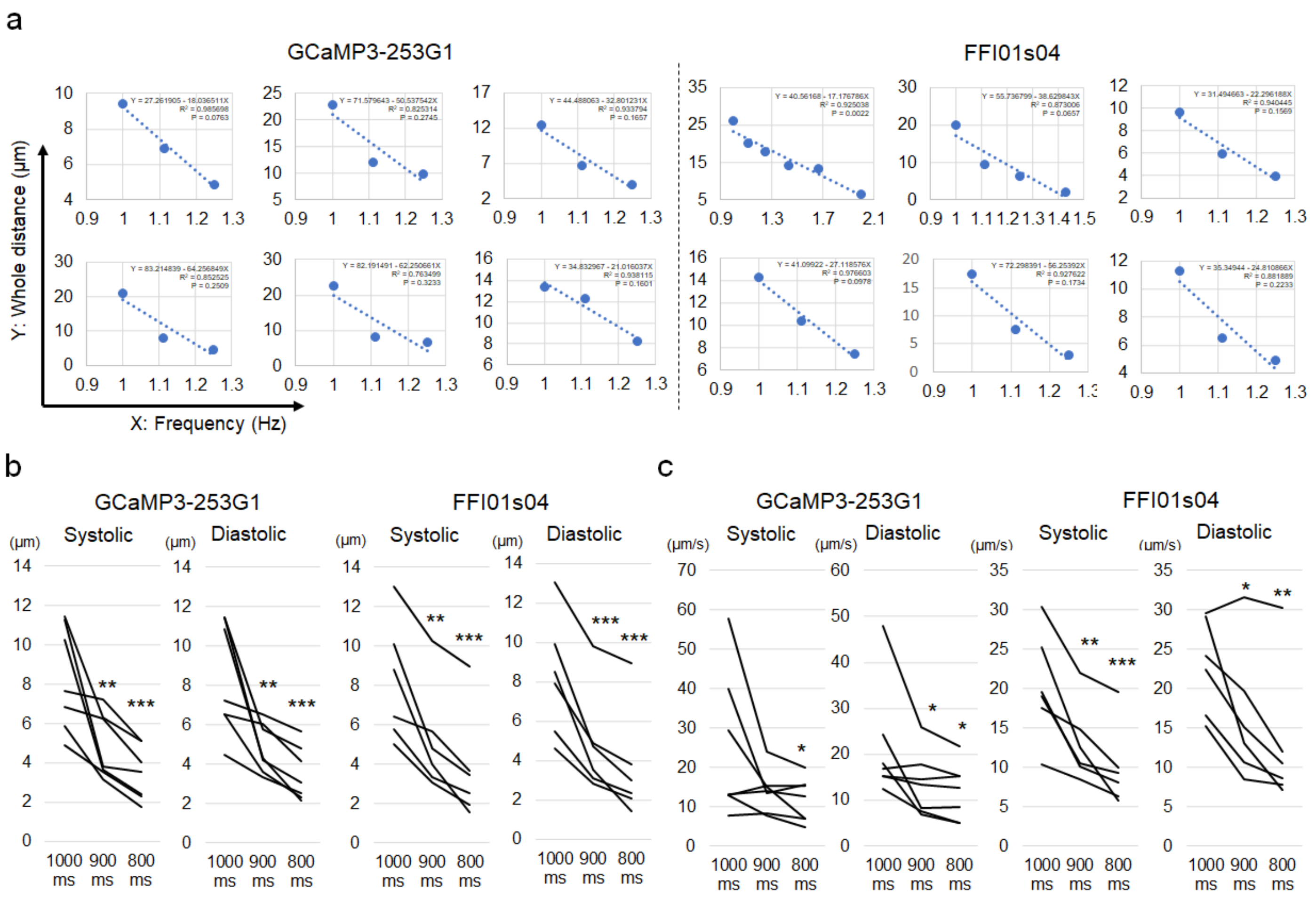

Supplementary Figure 2: Detection for particle displacement of HMD and analysis for tissue function.
(**a**) Relationship between frequency of electrical stimulation (X) and particle displacement distance in whole beating cycle (Y) in HMDs constructed from GCaMP3-253G1 and FFI01s04 human iPSC lines, respectively. Results of regression analysis are shown. (**b**) (**c**) Particle displacement distance and speed of systolic (**b**) and diastolic (**c**) phases at each electrical stimulation interval in HMDs constructed from GCaMP3-253G1 (n =7) and FFI01s04 (n =6) human iPSC lines, respectively. *P<0.05. **P<0.01. ***P<0.001.

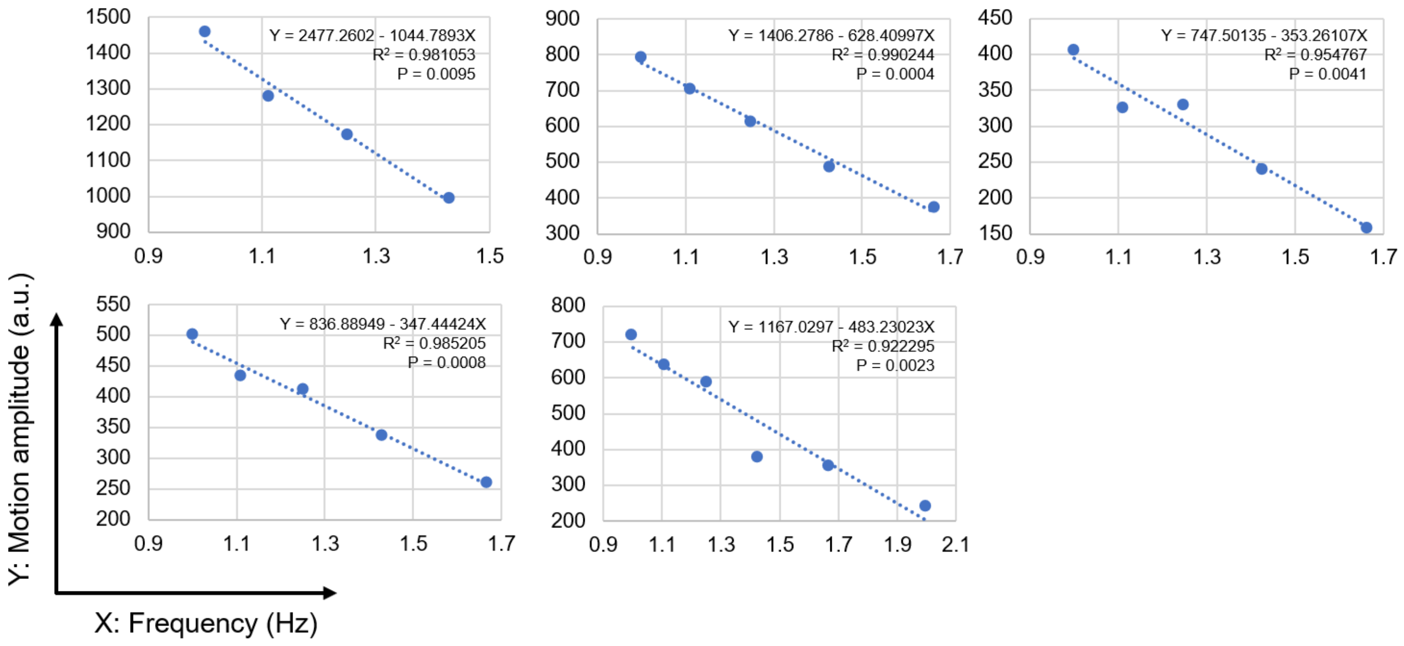

Supplementary Figure 3: MUSCLE MOTION analysis for 3D cardiac microtissues.

Relationship between frequency of electrical stimulation (X) and motion amplitude in whole beating cycle (Y). Results of regression analysis are shown.

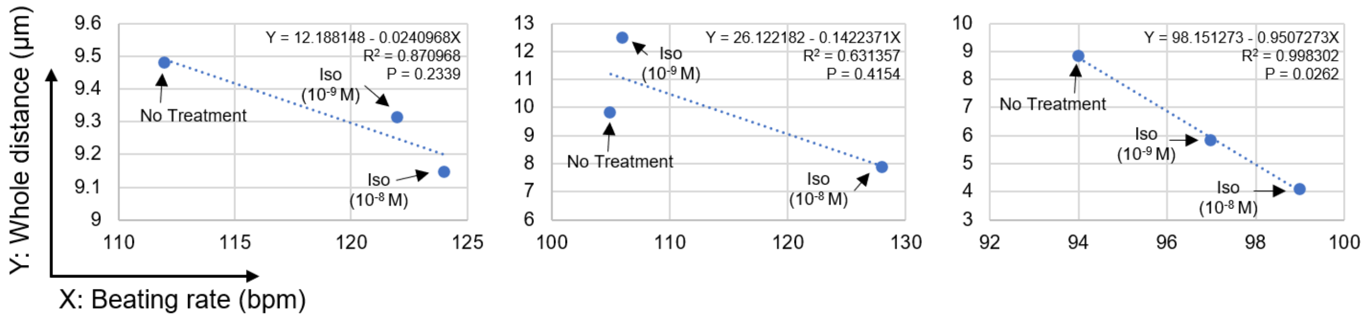

Supplementary Figure 4: Pharmacological responses of HMDs to isoproterenol.
Relationship between beating rate of HMD (X) and particle displacement distance in whole beating cycle (Y). Results of regression analysis are shown.

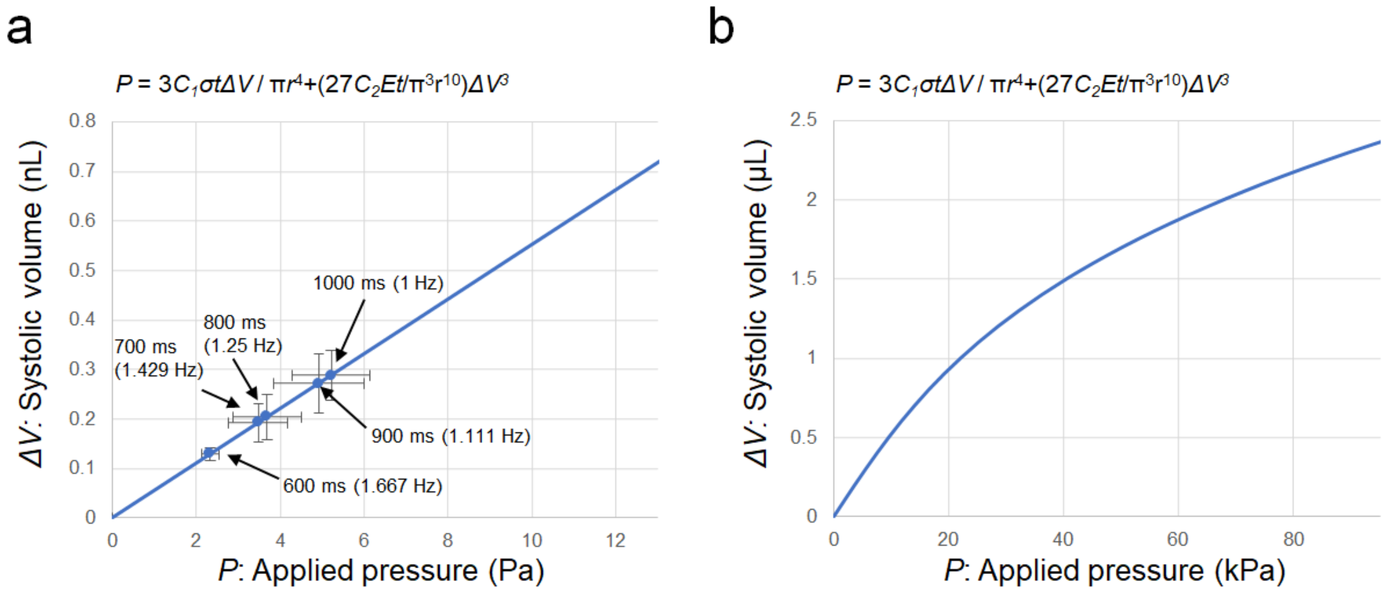

Supplementary Figure 5: A Frank-Starling curve calculated by HMD system.

The curves were calculated by an equation [$P = 3C_1\sigma t\Delta V / \pi r^4 + (27C_2Et/\pi^3 r^{10})\Delta V^3$] which was led by equations (2) and (3) in the main manuscript. *C1* and *C2* = constant depending on the diaphragm geometry and material, σ = residual stress of PDMS, t = diaphragm thickness, E = Young's modulus, r = radius of the chamber. (**a**) A curve calculated from a representative particle displacement according to the changes of frequency of electrical stimulation in the present study (correspondent to Fig. 3c). (**b**) A curve calculated with increased applied pressure and systolic volume. The range of systolic volume shown in the curve is based on theoretically maximal value of the systolic volume in the present design of microfluidic chip.
